# Tissue-specific aging: a tale of functional asymmetry

**DOI:** 10.18632/aging.100635

**Published:** 2014-01-30

**Authors:** Ismene Karakasilioti, George A. Garinis

**Affiliations:** ^1^ Institute of Molecular Biology and Biotechnology, Foundation for Research and Technology-Hellas, GR70013, Heraklion, Crete, Greece; ^2^ Department of Biology, University of Crete, Heraklion, Crete, Greece

Functional asymmetry is a fundamental aspect of all living beings. This also applies to single celled organisms that reproduce symmetrically into two identical halves; the cell that inherits the old pole, it grows at a slower rate, produces less offspring, and shows a decrease in overall cell viability (Stewart EJ et al., PloS Biol 2005; 2:e45). Functional asymmetry becomes clearly visible when one considers the dramatic differences between the immortal germ line and the disposable soma or between the countless cell types of multicellular organisms; most cells differ in their metabolic demands, replicative potential, and developmental origin or are exposed to distinct intrinsic and environmental hazards. However, what makes functional asymmetry more relevant for aging is that any unforeseen cellular mistake will also have asymmetric outcomes for human health e.g. the faulty repair of DNA lesions may have a much higher cost in one direction (e.g. stem cells) than in the other (e.g. hepatocytes). Moreover, functional asymmetry is frequently observed in pathways, such as those involved in more than one biological process; different mutations of the same gene or in different genes of the same pathway could impair one or the other function of that pathway triggering the onset of distinct pathological features.

Nucleotide excision repair is a conserved DNA repair pathway that cells employ to recognize and remove helix-distorting DNA lesions (Kamileri et al.; Trends Genet. 2012; 11:566-73). Defects in NER represent some of the best-known examples of functional asymmetry; the clinical outcome of NER patients is exceptionally diverse ranging from increased skin cancer predisposition (as in Xeroderma Pigmentosum) to a wide range of progeroid features (as in Cockayne syndrome or trichothiodystrophy) that once present, they manifest in some, but not all, organs. Recent work from our lab allowed us to gain further insights on how random DNA damage events could trigger the onset of tissue-specific pathological features in NER progeroid syndromes (Karakasilioti I et al. Cell metabolism. 2013; 18:3:403-415).

Using mice that lack the NER structure-specific endonuclease ERCC1 systemically or specifically in the adipose tissue, we found that the animals exhibited marked white and brown adipose tissue abnormalities. These appeared to be degenerative changes as both adipose tissue depots developed normally with defects gradually appearing only at later stages in murine life. Further work revealed that the accumulation of irreparable DNA inter-strand crosslinks triggers the transcriptional derepression of pro-inflammatory cytokines in adipocytes, the recruitment of macrophages to sites of tissue damage and the destruction of white adipose tissue depots in NER-defective animals. However, unless one considers the impact of functional asymmetry in age-related diseases, it is rather difficult to appreciate why the adipose tissue would be particularly sensitive to the NER defect. In this respect, a closer look at adipose tissue biology has proven valuable. The oxidation of fats and oils forms radicals capable of crosslinking DNA; in white adipose tissue, where lipids are most abundant, lipid peroxidation would further propagate the formation of irreparable DNA inter-strand crosslinks in ERCC1-defectice adipocytes. Moreover, the adipose tissue itself resembles an ancestral immune organ in many aspects; adipose lineage cells display macrophage properties, adipocytes secrete pro-inflammatory cytokines and the great majority of adipocytes that are located close to lymph nodes are known to interact with lymphoid cells (Caspar-Bauguil Set al., FEBS letters. 2005; 17:3487-3492). Taken together, the NER defect itself, lipid peroxidation and the inherent propensity of adipocytes to activate innate immune responses upon stress could establish self-perpetuating pro-inflammatory cycles. Evidently, the links existing between the adipose tissue and the immune system have evolved to allow injured cells to rapidly communicate their compromised state to the microenvironment; however, the accumulation of irreparable DNA lesions in NER-deficient animals or the gradual wear and tear of adipocytes with aging could establish a chronic inflammatory state leading to adipose tissue degeneration and, in turn, to systemic metabolic dysfunction.

